# Primary Cilia Structure Is Prolonged in Enteric Neurons of 5xFAD Alzheimer’s Disease Model Mice

**DOI:** 10.3390/ijms222413564

**Published:** 2021-12-17

**Authors:** Vu Thu Thuy Nguyen, Lena Brücker, Ann-Kathrin Volz, Julia C. Baumgärtner, Malena dos Santos Guilherme, Francesco Valeri, Helen May-Simera, Kristina Endres

**Affiliations:** 1 University Medical Center, Department of Psychiatry and Psychotherapy, Johannes Gutenberg University Mainz, 55131 Mainz, Germany; VuThuThuy.Nguyen@unimedizin-mainz.de (V.T.T.N.); bgt.j@web.de (J.C.B.); malena-guilherme@gmx.de (M.d.S.G.); francescovaleri47@gmail.com (F.V.); 2 Cilia Cell Biology, Institute of Molecular Physiology, Johannes Gutenberg University Mainz, 55131 Mainz, Germany; bruecker@students.uni-mainz.de (L.B.); avolz01@uni-mainz.de (A.-K.V.)

**Keywords:** Alzheimer’s disease (AD), primary cilia, enteric nervous system (ENS), ganglia, neurites

## Abstract

Neurodegenerative diseases such as Alzheimer’s disease (AD) have long been acknowledged as mere disorders of the central nervous system (CNS). However, in recent years the gut with its autonomous nervous system and the multitude of microbial commensals has come into focus. Changes in gut properties have been described in patients and animal disease models such as altered enzyme secretion or architecture of the enteric nervous system. The underlying cellular mechanisms have so far only been poorly investigated. An important organelle for integrating potentially toxic signals such as the AD characteristic A-beta peptide is the primary cilium. This microtubule-based signaling organelle regulates numerous cellular processes. Even though the role of primary cilia in a variety of developmental and disease processes has recently been recognized, the contribution of defective ciliary signaling to neurodegenerative diseases such as AD, however, has not been investigated in detail so far. The AD mouse model 5xFAD was used to analyze possible changes in gut functionality by organ bath measurement of peristalsis movement. Subsequently, we cultured primary enteric neurons from mutant mice and wild type littermate controls and assessed for cellular pathomechanisms. Neurite mass was quantified within transwell culturing experiments. Using a combination of different markers for the primary cilium, cilia number and length were determined using fluorescence microscopy. 5xFAD mice showed altered gut anatomy, motility, and neurite mass of enteric neurons. Moreover, primary cilia could be demonstrated on the surface of enteric neurons and exhibited an elongated phenotype in 5xFAD mice. In parallel, we observed reduced *β-Catenin* expression, a key signaling molecule that regulates Wnt signaling, which is regulated in part via ciliary associated mechanisms. Both results could be recapitulated via in vitro treatments of enteric neurons from wild type mice with A-beta. So far, only a few reports on the probable role of primary cilia in AD can be found. Here, we reveal for the first time an architectural altered phenotype of primary cilia in the enteric nervous system of AD model mice, elicited potentially by neurotoxic A-beta. Potential changes on the sub-organelle level—also in CNS-derived neurons—require further investigations.

## 1. Introduction

Primary cilia are microtubule-based appendages that extend from the cell membrane of virtually all post-mitotic eukaryotic cells. Although cilia are actually the oldest known cellular organelles, first described by Anthony van Leeuwenhoek in 1675, their critical role in a multitude of developmental and disease processes has only recently been recognized [1]. Not to be confused with motile cilia, which serve to generate fluid flow over membrane surfaces, primary cilia are specialized signaling organelles important for transduction of a wide range of signaling pathways [2]. Defects in ciliary signaling lead to a range of syndromic disorders collectively termed ciliopathies, which affect a multitude of organs and tissue types [3]. As research into primary cilia increases, it is becoming clear that ciliary defects also underlie a wide variety of non-syndromic diseases [4,5,6]. This is not surprising, given that primary cilia are so ubiquitous and play vital roles in cell and tissue homeostasis.

Cognitive impairment is a common feature associated with ciliary dysfunction, largely thought to be attributed to ciliary signal regulation in a multitude of neuronal cell types [7]. Very little is known about defective ciliary signaling and its contribution to neurodegenerative diseases such as Alzheimer’s disease (AD). The few studies that explored this association were focused on ciliary defects downstream of cellular pathogenesis [8,9,10]. Hu and colleagues were the first to describe a prolongation of cilia in the hippocampus of APP/PS1 mice in comparison to wild type controls [9], which subsequently affected the axonal growth of neurons. Moreover, activation of serotonin receptors was able to restore the ciliary malformation and by this, cognitive impairment of the animals. Since many ciliopathies are associated with cognitive disabilities, it is plausible to assume that primary cilia of the CNS might play a role in AD. However, the role of cilia on enteric neurons, the nerve cells of the gut, has not been considered yet. In recent years, the importance of the so-called brain–gut-axis in neurodegenerative diseases has been widely accepted [11]. Numerous investigations report on changes in gut microbiota and physiological properties of the gut in animal models and human patients. The occurrence of primary cilia in this cell type was first described in 2013 using neurons of rat duodenum myenteric plexus [12]. In line with this new gut-perspective on disorders that traditionally have been seen as purely affecting the brain, we wanted to investigate the impact of AD pathological hallmarks on primary cilia within the enteric nervous system (ENS). For this study, we examined 5xFAD mice [13] that carry three mutations in heterologous amyloid precursor protein (APP) and two in the Presenilin 1 (PS1) transgene which are responsible for the early onset of familial AD (FAD) and thereby display an aggressive version of AD-like pathology with phenotypes observed at the age of 1.5 months [14]. Changes in the gut microbiome [15,16] and in enzymatic and mechanistic function of the gut [15,17] have already been reported for this mouse model. Here, we show that besides micro-morphological alterations of the gut tissue, we observed defects in ENS cell ciliation, recapitulating the observations from the CNS. Furthermore, we also observed changes in downstream ciliary signaling pathways.

## 2. Results

### 2.1. Differences in Gut Anatomy and Function Driven by FAD Mutations in a Mouse Model of Alzheimer’s Disease

**Figure 1:**
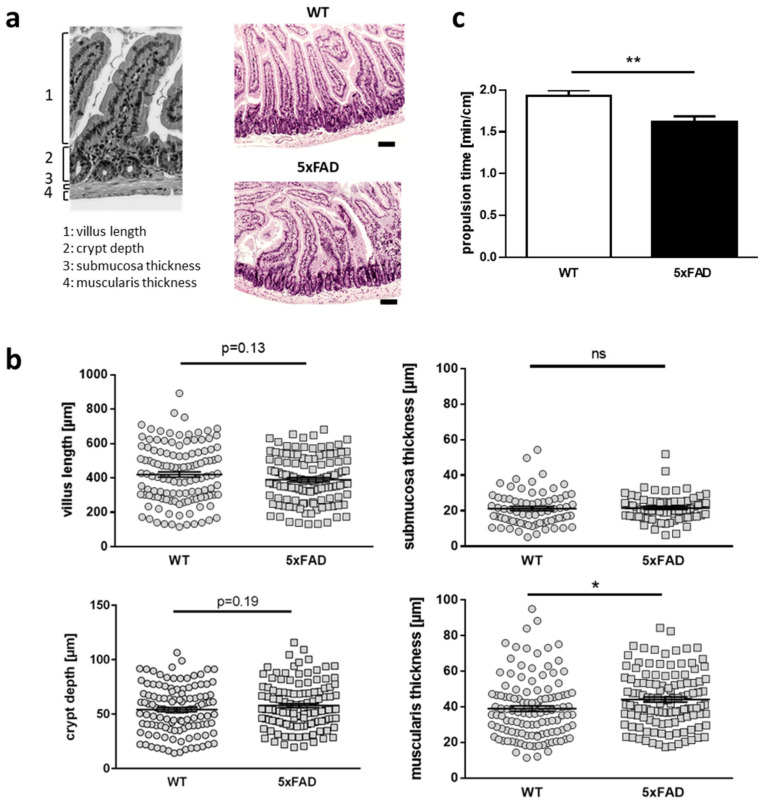
Morphological and functional changes were observed in the gut of 5xFAD model mice. (**a**) Quantitative parameters were assessed within duodenal slices of mice. Gut slices were stained with HE (representative images for wild type and 5xFAD mice are shown) and respective structures were measured (**b**) The four anatomical structures villus length, crypt depth, submucosa and muscularis thickness were analyzed with four measurements of each duodenal structure in three slides per mouse (*n* = 10 animals per group, aged 11 weeks, single values are shown) by using a clock-wise orientation in AD model mice and their respective wild type littermates. (**c**) Measurement of gut motility. Distal colonic sections (*n* = 6 per group, mean age 25 weeks) were dissected and inserted into an organ bath with carbogen-gas perfused Krebs solution. A fecal pellet was inserted in the oral end and time measured for transport to the anal end. Statistical analysis was performed by using Student’s *t*-test (*n* = 6 per group; * *p* < 0.05; ** *p* < 0.01).

Several publications have shown that the differences in body weight and microbiota depend on the lifetime state of mouse models of AD [15,18]. These findings suggest potential changes in gut functionality and/or morphology as mice mature or age. Puberty or adolescence presents a dynamic phase in general. We therefore measured several different parameters in duodenal sections of 11-week-old AD model mice and their respective wild type littermates. While villus length, crypt depth, and the submucosa thickness were not different to controls (Figure 1a,b), the height of the muscular layer was increased significantly in samples from mutant mice (*p* = 0.017). As an ex vivo surrogate for measuring gut function, we next assessed gut motility with isolated colonic stretches in an organ bath. The movement of fecal pellets revealed increased propulsion within the gut derived from transgenic mice (*p* = 0.008; Figure 1c).

In sum, our investigations revealed that gut functionality is affected in the AD mouse model not only at higher ages (25 weeks), but that we already observed impact on gut architecture, such as thickening of muscularis, very early on (11 weeks).

### 2.2. Induced Neurite Extension of Enteric Neurons Derived from 5xFAD Mice

**Figure 2:**
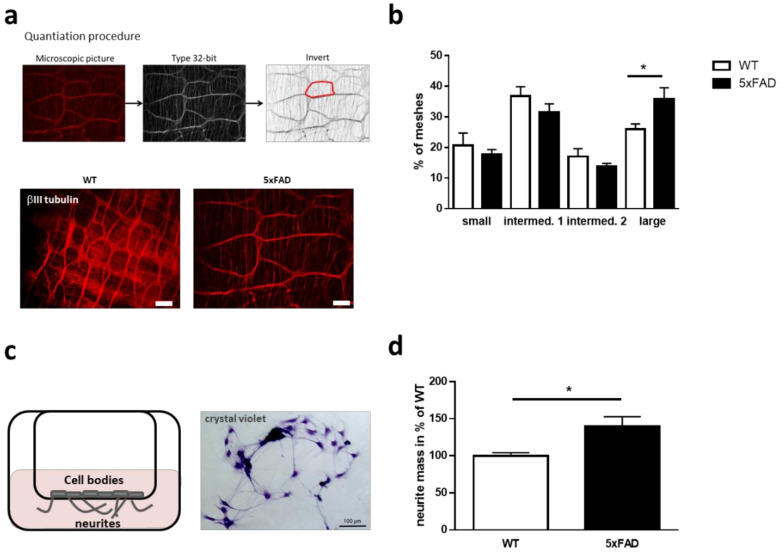
Primary enteric neuronal cultures of 5xFAD mice display increased neurite mass. (**a**) Preparations of myenteric plexus on longitudinal muscle (LMMP) were stripped off from colonic stretches and stained with βIII tubulin antibody. Procedure of quantitation is shown and one mesh area is labelled in red as an example. Representative pictures for wild type and 5xFAD are shown (scale bars: 100 µm). (**b**) Mesh area was calculated by ImageJ using two microscopic pictures per mouse. Meshes of the enteric network were subdivided in groups of 1500–4500 (small), 4500–10,000 (intermed. 1), 10,000–15,000 (intermed. 2), and 15,000–40,000 (large) µm^2^. Statistics were performed with One Way Anova (Fisher’s LSD test, *n* = 4 animals per group; aged 10–11 months, 10–36 meshes per animal; * *p* < 0.05). (**c**) Enteric neuron cultivation on transwell plates. Neurons were prepared from myenteric plexus and seeded on 3 µm transwell inserts. A representative microscopic picture of crystal violet stained neurons is shown. Neurites are seen as thin protrusions between cells or cell clusters. (**d**) On DIV4, cells were stained with crystal violet, cell bodies removed, neurite-attached stain extracted, and stain intensity measured. Cultures derived from 4 independent donor mice were analyzed with technical triplicates (*n* = 12 per group). Values were normalized to values obtained for wild type and are presented in percentage as mean + SEM. Statistical analysis was performed by using Student’s *t*-test with Welch correction (* *p* < 0.05).

Muscular strength is not the only mechanism to regulate peristalsis of the gut, neuronal signaling from the ENS is also involved [14]. To investigate whether the enteric neuron network in our mutants was affected, we measured the neuronal network architecture. For this, enteric neurons of the colonic myenteric plexus were visualized via immunohistochemistry using an antibody against βIII tubulin. Afterwards, the size of meshes was assessed (Figure 2a,b). Mean size of neuronal network meshes was not affected (data not shown, *p* = 0.11); however, closer investigation of mesh-subtypes did reveal changes: smaller relative mesh amounts were reduced, albeit non-significantly, while larger sized meshes were significantly increased (*p* = 0.014, Figure 2b).

To analyze whether single enteric neurons isolated from these mutant mice also exhibit changes regarding cellular structure, we investigated their capability to extend neurites from the cell body. For this, cells were seeded with the same cell numbers for both conditions on a transwell system and neurite outgrowth was determined after four days in culture (DIV4). Crystal violet staining (Figure 2c) showed a significantly increased neurite mass of up to 140% in primary cells from mutant mice, compared to wild type littermates (*p* = 0.012, Figure 2d). This means that not only the muscularis is changed due to the AD genotype but also connectivity parameters of the ENS—at least in older mice. 

### 2.3. Characterization of Primary Cilia in Primary Enteric Neuron Culture of 5xFAD Mice

**Figure 3:**
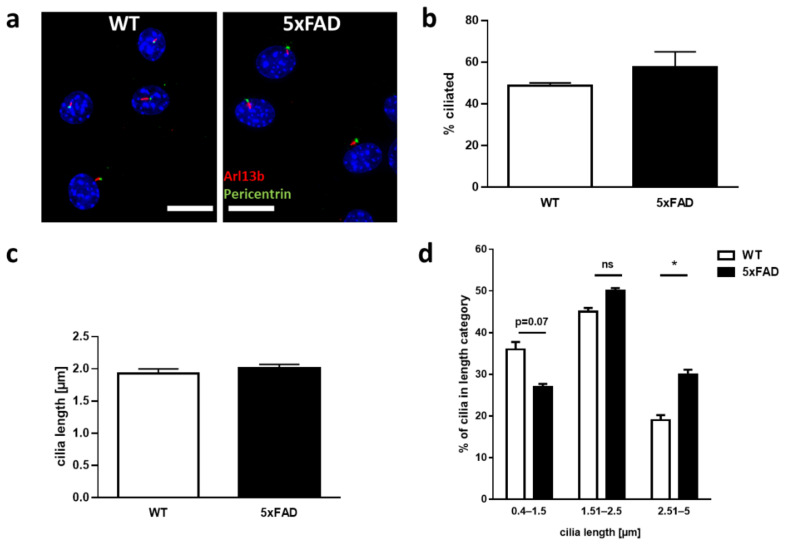
Higher prevalence of elongated primary cilia in 5xFAD-derived enteric neurons. (**a**) Staining of cilia. Neurons were prepared as described before and seeded on cover slips. A representative microscopic image of neurons after staining for the ciliary membrane protein Arl13b (red) and the basal body marker Pericentrin (green) and counterstain with DAPI (blue) of both genotypes is shown (scale bars: 10 µm). (**b**) Quantification of ciliated cells. Number of cells carrying cilia was assessed and is presented in% of value obtained for wild type controls. The percentage of ciliated cells was calculated by determination of DAPI stained cells that showed Arl13b- and Pericentrin-double labelled primary cilia. Cultures derived from 4 independent donor mice in each group were analyzed with technical triplicates from 20 random visual fields with an average of 8 cells per image. (**c**,**d**) Cilia length was measured with ImageJ and mean length (**c**) or distribution in regard to length groups (**d**) was shown. Cultures derived from 3 (WT) and 4 (5xFAD) independent donors were analyzed with technical duplicates (*n* = 159 cilia measured for WT and 260 for 5xFAD). Data are presented as mean + SEM. Statistical analysis was performed by using Student’s *t*-test or One Way Anova (post-test Fisher LSD; * *p* < 0.05).

The primary cilium is an important cellular structure, required for a range of cellular processes, in particular sensing external signals and regulating signaling pathways. Such processes affect cellular morphology, including neurite outgrowth. Since neurite outgrowth was changed due to the FAD genotype, we sought to characterize primary cilia in the neuronal cultures. For this, immunohistochemistry on enteric neurons was performed using antibodies against the ciliary membrane (Arl13b) and the basal body at the base of the cilium (Pericentrin) (Figure 3a). We chose six-month-old mice for this purpose, as this presents an age where changes regarding cilia of CNS neurons have already been reported [19] and where changes in gut morphology are already observed but no massive neuronal loss in the ENS is to be expected. The percentage of ciliated cells, as well as ciliary length, was determined in enteric neuronal cultures from mutant and littermate controls (Figure 3b–d). No significant differences could be observed in terms of ciliation, although a slight trend towards increased ciliation could be seen in mutant cultures (*p =* 0.35). Analysis of mean ciliary length also showed no significant difference between mutant and control (*p =* 0.5); however, when the measured cilia length was stratified into short (0.4–1.5 µm), medium (1.51–2.5 µm), and long (>2.5 µm), we observed a significant increase in longer cilia and fewer shorter cilia in 5xFAD enteric neurons (*p =* 0.027, Figure 3d).

Our results confirmed the occurrence of primary cilia on enteric murine neurons and demonstrated an increase in the proportion of longer cilia in enteric neurons of the colon derived from 5xFAD mice.

### 2.4. Effects of Acute Administration of A-beta Peptide on Primary Cilia of Murine Enteric Neurons and on Ciliary Signaling Pathways

The increased number of elongated primary cilia in enteric neurons cultured from 5xFAD mice might be attributable to heterologous expression of mutated APP or PS1, increased production of neurotoxic A-beta peptide or due to secondary events such as elevated ROS production or inflammatory signals. To decipher a possible underlying mechanism, we incubated wild type enteric neurons with either recombinant A-beta_42_ or a scrambled negative control peptide devoid of biological function. A-beta can interact with potential receptors and activate downstream pathways to generate reactive oxygen species, hyperphosphorylated tau protein, and cause inflammatory responses [20]. To mimic one of the downstream effects, Rotenone—an inducer of ROS production—was administered. Cilia were stained as before, and their characteristic features were determined as described above.

**Figure 4:**
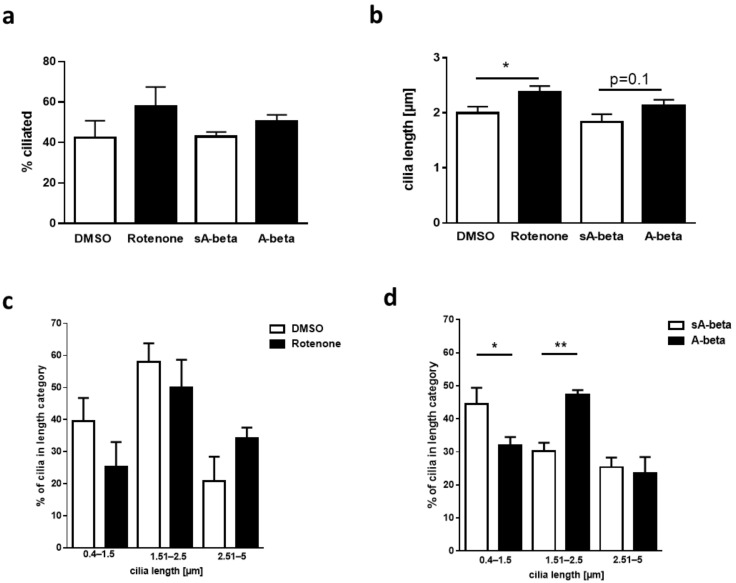
Impact of acute A-beta administration on cilia length of enteric neurons. (**a**) Enteric neurons (DIV3) of wild type mice were treated for 24 h with scrambled peptide (sA-beta), A-beta_42_ (both: 2.5 µM) or Rotenone (0.5 µM). Cilia characteristics were determined as described in Figure 3 (*n* = 76 cilia measured after treatment with DMSO, *n* = 120 with Rotenone, *n* = 58 with sA-beta and *n* = 118 with A-beta). (**b**) Cilia length after treatment. Statistical analysis was conducted using Student’s *t*-test. Group-stratified mean cilia length after treatment with Rotenone (**c**) or with peptides (**d**). One Way Anova (post-test Fisher LSD; * *p* < 0.05, ** *p* < 0.01).

Neither Rotenone nor A-beta_42_ had a significant impact on the percentage of ciliated cells (Figure 4a). However, a slight increase of mean cilia length was observed for both treatments (Figure 4b), with a statistical significance upon treatment with Rotenone with a cilia length of 2.4 µm (*p* = 0.024). Surprisingly, closer examination of ciliary size distribution, showed that treatment with Rotenone lead to non-statistical changes towards longer cilia (>2.5 µm; Figure 4c). On the contrary, A-beta_42_-administration showed a significantly reduced percentage of shorter cilia (*p* = 0.0254) with a significantly increasing number of medium length cilia (1.51–2.5 µm; *p* = 0.0064, Figure 4d).

While the Rotenone treatment lead to non-statistical changes, we observed that acute A-beta administration on wild type derived enteric neurons affected cilia length with an increasing number of medium length cilia.

**Figure 5:**
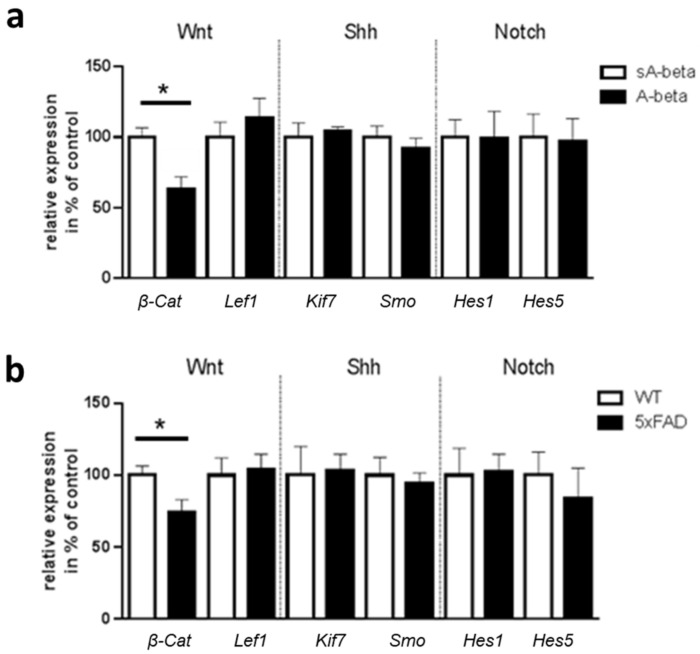
Effect of acute A-beta administration or of chronic exposure within transgenic enteric neurons on ciliary signaling pathways. (**a**) Enteric wild type neurons (DIV3) were treated for 24 h with A-beta peptides or scrambled control (sA-beta). mRNA was used for analysis of depicted gene products with *Gapdh* mRNA levels for normalization (*n* = 6–8 per group). (**b**) Colonic mRNA of 8-month-old mice (WT or 5xFAD) was subjected to qPCR analysis. mRNA levels obtained were normalized to *Gapdh* mRNA levels (*n* = 5–6 per group). Statistical analysis was performed by pairwise *t*-tests (* *p* < 0.05). All data are presented as mean + SEM.

Primary cilia are required for the regulation of numerous signal transduction pathways, for example Wnt, Shh or Notch signaling [21], many of which are regulated in a cell type specific manner. Since ciliary changes were observed in enteric neurons with acute A-beta treatment as well as those cultured from the 5xFAD model mice, we analyzed mRNA levels of central players of these pathways (Figure 5).

We only saw differences in gene expression of *β-Catenin*, which is a downstream target of canonical Wnt signaling. Upon activation of canonical Wnt, β-Catenin enters the nucleus and, in cooperation with the transcription factor Lef1, activates the transcription of Wnt target genes. A reduced *β-Catenin* mRNA level was found in both, acutely treated neurons (*p* = 0.024, 63% of sA-beta control) and colonic tissue of 5xFAD mice (*p =* 0.046, with 74% of wild type controls). However, downstream Lef1 expression levels were not changed, suggesting an alternative regulation. No differences were observed for mRNA levels of Shh components *Smo* and *Kif7* or *Hes1* and *Hes5*, both target genes of Notch, indicating no impact on these ciliary signaling pathways in AD pathology affected enteric neurons.

In summary, no changes for Shh and Notch signaling were detected, whereas the expression level of *β-Catenin* was reduced in enteric neurons of the 5xFAD transgenic mice and also in wild type enteric neurons after acute A-beta administration.

## 3. Discussion

The relevance of the gut–brain-axis for neurodegenerative diseases has become an intense research topic within the last few years (reviewed in [22]). For instance, differences in gut anatomy have been described for many rodent models of diseases such as AD or Parkinson’s disease (PD) [16,23]. It has been suggested that α-synucleinopathy of the ENS could be an early indicator of PD pathology [24]. In AD, the observed expression of APP in the ENS supports the supposition of the involvement of the intestinal nervous system in this disease [25]. The presence of primary cilia in differentiated neurons of the enteric nervous system has been described so far only once [12]. Nevertheless, the role of this highly dynamic organelle in the neurons of the ENS and identification of the consequences of its dysfunction has not been investigated comprehensively. Its association with neurodegenerative diseases is only now starting to be elucidated but mostly with a focus on cells of the central nervous system (reviewed in [26]). Therefore, we set out to examine the ciliary phenotype in enteric neurons of the widely used 5xFAD Alzheimer mouse model in an attempt to gather novel insights into possible gut-prone disease mechanisms. Our study demonstrates changes in gut anatomy, motility, and neurite growth from enteric neurons in 5xFAD mice as compared to wild type littermates. It has already been shown that the gut–brain-axis is relevant for neurodegenerative diseases. The thickening of the muscular layer that were assessed might be a consequence of inflammatory processes (e.g., shown in Crohn’s disease, [27]). Inflammation is a central mechanism in AD with presence of a sustained immune response in the brain [28]. A significant increase in glial fibrillary acidic protein (GFAP) expression, one of the major indicators of inflammation, was also observed in the colon of 5xFAD animals as compared to same-age littermates between 8 and 40 weeks of age [17]. Since layers of smooth muscles are major prerequisite for peristalsis (rhythmic waves of contractions), an impact of such tissue thickening on peristalsis is expected in this regard [29]. In fact, a decreased gut transit time was observed for 5xFAD mice resulting in a faster feces expelling time [17]. This is consistent with our observation that ex vivo, colonic propulsion increases in 5xFAD model mice. Neurons, which are embedded within the ganglia that form the myenteric and submucosal plexus of the ENS, control gastrointestinal behavior including secretion, but also motility [30,31]. Changes in the architecture of the ENS therefore obviously will have an impact on the function of the gut. In the extreme, this is found for Hirschsprung disease, where lack of colonic neurons impairs normal function severely [32]. In the 5xFAD mice, a higher proportion of larger meshes of ENS was found. Similar differences in enteric network density of APP transgenic mice were also shown for TgCRND8 mice before [33]. A reason for the observed impoverishment of the neuronal network might rely on altered neurite outgrowth. An increase in neurite outgrowth was measured in neurons of the ENS derived from 5xFAD mice as compared to wild type. Whether the alterations observed are related to changes in neurite length, neurite number or a combination of both, remains to be determined as here, neurite mass as a sum parameter was measured. As the method requires removing cell bodies, we were not able to assess potential differences in proliferation or growth properties that might have affected neurite mass, even if we initially seeded the same amounts of cells. However, Mazi and colleagues already revealed an increase in the neurite number of 5xFAD mouse neurons derived from the cortex, hippocampus, and cerebellum as compared to their non-transgenic littermates [34]. In another in vitro study, treatment of rat pheochromocytoma PC12 cells with membrane associated and soluble human brain APP resulted in enhanced neurite outgrowth by increasing neurite length and number of branch points per cell [35]. This coincides with our data from 5xFAD enteric neurons. Probably, intensified growth of neurites hampers the organization of mesh contact sites. However, the underlying signaling pathways causative for this observation still need to be elucidated.

An organelle majorly involved in the perception of external signals in many organs and tissues is the primary cilium [21]. The presence of primary cilia on enteric neurons has only been demonstrated in one report so far by transmission electron microscopy [12]. Here, we confirmed the occurrence of cilia on primary cultures of enteric murine neurons by immunostaining of characteristic marker proteins such as Arl13b. While in our study the average cilia length and number of ciliated enteric neurons of the colon derived from 5xFAD mice was unaltered in comparison to wild type mice, we did observe an increase in the proportion of longer cilia. Luesma and colleagues described primary cilia in the myenteric plexus of the rat duodenum using electron microscopy with the mean neuronal cilium length between 2 to 4 μm [12]. This is consistent with the measured length of primary cilia in our study. Comparatively, the primary cilia length in the central nervous system varied across brain regions, ranging from 2.1 to 9.4 μm [12]. The detailed role of primary cilia present in different neuronal cell types is still under investigation. For example, conditional ablation of primary cilia has been shown to result in disrupted dendritic arborization and the disturbed establishment of neuronal networks of adult-born hippocampal neurons in C57BL/6 mice [36]. Genetically modified mouse models with disrupted primary cilia showed the role of the organelle for neurodevelopment and maintenance of neurogenesis in adult hippocampal and cortical structures [37,38]. While the importance of primary cilia is apparent with the emergence of diseases directly associated with the organelle, collectively termed ciliopathies, its role in the enteric nervous system as well as in AD has not been analyzed before. In vitro the A-beta exposure of murine NIH3T3 fibroblasts resulted in significant reduction in primary cilia length [10]. The only two reports on changes of primary cilia length in CNS neurons from AD mouse models available so far revealed contradictory results [9,39]. Hippocampal dentate granule cells from 3xTg-AD mice displayed shortened primary cilia [39]. By contrast, cilia of 2xTg-AD mice in the same study were as long as the wild type cells’ cilia. These results show different outcomes dependent on the respective mouse strain. While the 2xTg-AD model harbors APP and PS1 transgenes, the 3xTg-AD additionally has mutated Tau, which might explain the different results obtained. In another study, conducted on 3-month-old APP/PS1 mice [9], the primary cilia of hippocampal neurons were elongated as visualized by immunostaining. This is consistent with our observation of a significant increase in longer cilia (>2.5 µm) in enteric neurons of 5xFAD mice. A higher amount of medium-long cilia (1.51–2.5 µm) occurred in wild type neurons treated with A-beta peptides in vitro. The slight shift in the distribution of cilia length could be based on the comparison of an intrinsic system, where A-beta exposure is introduced piecewise throughout the life of the mice to an exogenous administration of A-beta, where changes could be due to the treatment period and concentration.

We wanted to mimic one of the consequences of A-beta treatment by administration of Rotenone which increases ROS production. Since this only represents one of the downstream effects of A-beta the obtained differences in the distribution of cilia length after Rotenone and A-beta treatment can be explained.

Generally, elongation of primary cilia would be likely to increase their sensory capacity because of the higher number of available receptors for signaling on the surface [2]. This might allow better adaptation to environmental changes [40], but also might result in an increased vulnerability towards noxes in the diseased state. A longer cilium might also be an indicator for defective ciliary disassembly, which is often observed in ciliopathies as well. The changes in ciliary length, could mean that this has a knock-on effect on its sensory capacity. It could be that changes in ciliary trafficking, ultimately affect the observed changes in ciliary length, thereby being a cause and not a consequence for ciliary defects. 

Since primary cilia contain a variety of receptor proteins [2] and are necessary for specific signaling cascades such as the Wnt signaling pathway, Notch signaling, and Shh pathway [21,41], we investigated mRNA level of central players of these pathways in wild type enteric neurons acutely treated with A-beta and in neurons of 5xFAD model mice. No changes for Shh and Notch signaling were observed, whereas the expression level of *β-Catenin* was reduced in enteric neurons of the 5xFAD transgenic mice and also in wild type enteric neurons after acute A-beta administration. β-Catenin is a key molecule in the canonical Wnt signaling pathway which is an essential signal transduction pathway that regulates numerous cellular processes such as neuronal survival and neurogenesis [42]. Altered ciliary signaling affecting downstream signaling pathways results in transcriptional regulation of targets, e.g., *β-Catenin*. Moreover, dysregulated Wnt/β-Catenin signaling plays an important role in the pathogenesis of AD. Jia and colleagues showed that Wnt/β-Catenin signaling is suppressed in brain of AD mouse models and an activation of Wnt/β-Catenin signaling inhibited A-beta production and tau protein hyperphosphorylation in the brain [43]. The connection between ciliary abnormalities and disrupted Wnt signaling in enteric neurons warrants further detailed examination in subsequent studies. However, other signaling systems should not be neglected. For example, a recent avenue of research is the role of somastatin in ciliary function since primary cilia express the somastatin receptor subtype SSTR(3), which is distinct from other neuronal compartments [44]. Studies have shown a downregulation of SST in AD [45]. A study using 6- to 8-month-old 3xTg-AD mice, producing both, A-beta_1-42_ and the mutant human tau protein, showed significantly shorter SSTR(3)-bearing primary cilia in hippocampal dentate granule cells compared to the corresponding wild type mice [39].

The presence of somastatin in the ENS [46] reveals this as an interesting starting point for future investigations. Furthermore, cilia to nuclei ratio of p75 neurotrophin receptor (p75NTR)- and SSTR(3)-loaded cilia was significantly higher in the dentate gyrus of 3×Tg mice as compared to wild type [19]. In the future, this signal pathway has to be further investigated for the potential role of the primary cilium in AD pathology.

Here, we show changes of the gut throughout the lifespan with a closer look at the primary cilia in the enteric nervous system of AD model mice. Only a few studies report on the connection between ciliopathies and AD in the ENS. In a zebrafish cilia model using *bbs8* morphants, a lack of enteric neurons resulting in gut motility defects was observed, showing the role of a ciliary protein in normal gut functioning [47]. We could reveal for the first time an architectural altered phenotype of primary cilia, elicited potentially by neurotoxic A-beta in six-month-old mice. To conclude whether our observation of changes are the cause for AD pathology or secondary consequences further investigations are needed. For example, mice at an age of onset of the disease should be examined for ciliary changes. Furthermore, additional research is necessary to determine particular signaling pathways affected by altered cilia structure in AD. 

In summary, our investigation provides further evidence that a direct impact of the disease-driving transgenes is present in the gut of genetic AD model mice, influencing its morphology and function. Moreover, our study emphasizes that primary cilia and ciliary signaling might play a role in AD pathogenesis as a new finding in this research field.

## 4. Materials and Methods

### 4.1. Animals

Male 5xFAD mice (APP K670N, M671L, I716V;PS1 M146L, L286V; Jackson Laboratory, Bar Harbor, ME, USA) were crossbred for maintenance with female C57B/6J mice (animal facility of the University Medical Center of Mainz). Food (Mouse breeding extrudate, ssniff Spezialdiäten GmbH, Soest, Germany) and water were provided *ad libitum* and mice were kept under a 12 h light/dark cycle. All experimental procedures were carried out in accordance with the European Communities Council Directive regarding care and use of animals for experimental procedures and approved by local authorities.

### 4.2. HE Staining and Duodenum Morphological Analysis

Duodenum samples from wild type and 5XFAD mice (aged 11 weeks) were immediately collected after dissection and fixed in 4% paraformaldehyde for 24 h at RT. Samples were embedded with paraffin and cut transversally into 2.5 µm thick sections (three per mouse). The sections were incubated at 60 °C for 30 min, dewaxed in xylene and subsequently hydrated by descending grades of alcohol (Ethanol 100%, 96% and 70%). The sections were stained with haematoxylin and eosin (H&E) according to standard procedure, dehydrated by ascending grades of alcohol (ethanol 70%, 96% and 100%), and cleared with xylene. Three pictures were captured per mouse using EVOS XL Core microscope (ThermoFisher Scientific, St. Louis, MO, USA) at 4× magnification. Morphological parameters such as crypt depth, villus length, muscular layer, and submucosa thickness were analyzed by ImageJ software (Rasband, W.S., ImageJ, Bethesda, MD, USA). Four villi and crypts length measurements were conducted in the same position for every duodenum picture and the thickness of submucosa and the muscular layer were analyzed strictly below the crypts. All morphological analyses were performed blinded regarding the genotype.

### 4.3. Ex Vivo Gut Motility Measurement

The apparatus used for this experiment was modified from [48]. The proximal colon was dissected from the mouse and fecal content was removed with pre-warmed Krebs solution (NaCl, KCl, NaH_2_PO_4_∙2H_2_O, MgSO_4_∙7H_2_O, NaHCO_3_, CaCl_2_∙2H_2_O, and Glucose). The gut was fixated in the organ bath with the oral end directed towards the influx site of the Krebs solution (37 °C and carbogen gas-treated). A small degree of laxity was considered to allow the segment to move freely 1 cm from the middle. A dried fecal pellet was inserted into the oral end of the colon. The pellet movement was recorded and the rate of fecal pellet propulsion determined.

### 4.4. Primary Culture of Enteric Neurons

Enteric neurons were obtained for primary culture from 6-month-old mice following the protocol of [49]. The abdominal skin and cavity were opened to reveal the internal digestive organs. The gut was dissected out and flushed with ice-cold Krebs solution. The longitudinal muscle containing the myenteric plexus was opened by removing the attached mesenteria. For isolation of the longitudinal muscle, it was teased away from the circular muscle with a cotton swab using horizontal strokes. The tissue was digested at 37 °C and 300 rpm for 60 min using Collagenase Type 2 (1.3 mg/mL, Worthington Biochemical Corporation, Lakewood, NY, USA). Cells were collected by centrifugation and filtered through a 100 µm cell strainer (Greiner bio-one). Cells were then diluted and maintained in culture medium (Neurobasal A, supplemented with 10 ng/mL GDNF (Origene), 1x B-27, 1% FBS, 2 mM L-Glutamine, and 1× Penicillin-Streptomycin (all Life Technologies, Carlsbad, CA, USA)) for four days at 37 °C, 5% CO_2_ and 95% air humidity. Quantification of the neurite outgrowth of the enteric neurons was assessed as described below. 

### 4.5. βIII Tubulin Staining of LMMPs

For investigation of mesh sizes of myenteric plexus in 11-month-old mice, colon LMMPs were isolated and fixed for 20 min in 4% PFA at RT. LMMPs were washed once with 1x PBS and blocked for 1 h at RT with Immunofluorescence Blocking Buffer (Cell Signaling, Danvers, MA, USA). Primary antibody β3-Tubulin (D71G9) XP^®®^ Rabbit mAb (Cell Signaling, Danvers, MA, USA) was incubated overnight at 4 °C. Conjugated secondary antibody Alexa Fluor 594 was incubated for 1 h at RT. Immunolabelling was imaged on ZOE™ Fluorescent Cell Imager (Bio-Rad, Hercules, CA, USA). 

### 4.6. RNA Preparation and qPCR

RNA was extracted from enteric neurons [17]. Quantitative polymerase chain reaction (qPCR) was performed using exon–exon boundary-spanning primer sequences (Table 1) and the SYBR Green methodology on a Step One Plus sequence amplification system (Applied Biosystems, Foster City, CA, USA). The relative mRNA expression of the tested gene normalized to *Gapdh* expression was calculated using the ΔΔCt method.

### 4.7. Neurite Mass Quantitation

Quantification of the neurite outgrowth of the enteric neurons was conducted using a cellular transwell culturing system modified from [50]. The cell culture transwell inserts with 3 micron pore size in 24-well formats (NuncTM cell culture inserts in multi-well plate) were coated with extracellular matrix gel from Engelbreth-Holm-Swarm murine sarcoma (Sigma Aldrich, diluted 1:5 with culture medium, St. Louis, MO, USA). After an incubation period of 24 h, neuronal cell bodies from the upper surface of the insert were removed with a cotton swab. Neurites were subsequently stained with crystal violet solution (Sigma Aldrich). For extraction of the dye, inserts were transferred into 400 µL extraction buffer and shaken for 5 min at 600 rpm at RT. To quantify neurite outgrowth, 100 µL of extracted stain solution was measured at OD595 and normalized to OD340 (Biochrom ASYS Hitech Expert 96 UV Microplate Reader).

### 4.8. Immunofluorescence

ENS cultures of 5XFAD and wild type littermates were fixed with 4% paraformaldehyde (PFA) for 20 min followed by formaldehyde quenching with 50 mM NH_4_Cl for 10 min. Cells were permeabilized with PBSTx (0.3% TritonX) for 15 min and blocked for 1 h at RT with FishBlock (0.1% Ovalbumin, 0.3% TritonX, 0.5% Fish gelatine in PBS). Primary antibodies for the centrosomes (Rb anti-pericentrin, Abcam ab4448, 1:500) and the ciliary membrane (Mm anti-Arl13b, Abcam ab136648; 1:200) were diluted in FishBlock and incubated overnight at 4 °C. Conjugated secondary antibodies Alexa Fluor 488 and 555 (1:400; Molecular Probes) and DAPI (Invitrogen) were incubated for 1 h at RT before mounting the coverslips on superfrost slides with Fluoromount-G^®®^ (SouthernBiotech, Birmingham). Immunolabelling was imaged on a Leica DM6000B fluorescent microscope (Leica, Bensheim, Germany). Deconvolution (BlindDeblur Algorithm, one iteration step) and processing of the images was performed with the Leica imaging software LAS X. Cilia length measurements were performed blinded regarding treatment or genotype using Fiji/ImageJ software (NIH, Bethesda, Rockville, MD, USA). A cilium was counted if a double staining of Arl13b and Pericentrin was visible and the length was measured via Arl13b signal in double stained cilia.

### 4.9. Statistics

Statistical significance was tested with One-way ANOVA followed by the indicated post-test or by unpaired Student’s t test when appropriate (GraphPad Prismversion 6 and 8, San Diego, CA, USA). Values of *p* < 0.05 were considered statistically significant.

## Figures and Tables

**Table 1:** Primer sequences.

Target Gene	Primer	5′-3′
*β-Catenin*	forward	ACTGCTGGGACTCTG
	reverse	TGATGGCGTAGAACAG
*Lef1*	forward	GTCCCTTTCTCCACCCATC
	reverse	AAGTGCTCGTCGCTGTAG
*Kif7*	forward	CTGGAGAAGGACTAGGTCG
	reverse	TTTCCAGGCAGACGCTTCTC
*Smo*	forward	GCTGCCACTTCTATGACTTCT
	reverse	GCCGATTCTTGATCTCACAGT
*Hes1*	forward	CGAGCCTGTTGGGGAAATAC
	reverse	GGTAGGTCATGGCGTTGATC
*Hes5*	forward	CTGGTGCAGGCTCTTGG
	reverse	AAACAAGTACCGTGGCGGTGGA
